# The blockage of the Nogo/NgR signal pathway in microglia alleviates the formation of Aβ plaques and tau phosphorylation in APP/PS1 transgenic mice

**DOI:** 10.1186/s12974-016-0522-x

**Published:** 2016-03-03

**Authors:** Yinquan Fang, Lemeng Yao, Chenhui Li, Jing Wang, Jianing Wang, Shujian Chen, Xin-fu Zhou, Hong Liao

**Affiliations:** Jiangsu Key laboratory of Drug Screening, China Pharmaceutical University, 24 Tongjiaxiang Street, Nanjing, 210009 China; School of Pharmacology and Medical Sciences, University of South Australia, Adelaide, SA 5000 Australia

**Keywords:** Nogo, NgR, AD, Microglia, Neuron, Aβ, Tau, Neuroinflammation

## Abstract

**Background:**

Alzheimer’s disease (AD) is characterized by extracellular β-amyloid (Aβ) plaques, neurofibrillary tangles (NFTs), and microglia-dominated neuroinflammation. The Nogo/NgR signal pathway is involved in AD pathological features, but the detailed mechanism needs further investigation. Our previous studies have confirmed that the activation of NgR on microglia by Nogo promotes the expression of proinflammatory cytokines and inhibits cell adhesion and migration behaviors. In the present study, we investigated the effects of Nogo/NgR signaling pathway on the pathological features of AD and possible mechanisms.

**Methods:**

After NEP1-40 (a competitive antagonist of Nogo/NgR pathway) was intracerebroventricularly administered via mini-osmotic pumps for 2 months in amyloid precursor protein (APP)/PS1 transgenic mice, plaque load, tau phosphorylation, and inflammatory responses were determined. After primary mouse neurons were exposed to the conditioned medium from BV-2 microglia stimulated by Nogo, the production of Aβ and phosphorylation of tau was quantified by ELISA and western blot.

**Results:**

Inhibition of the Nogo/NgR signaling pathway ameliorated pathological features including amyloid plaques and phosphorylated levels of tau in APP/PS1 mice. In addition, after treatment with the conditioned medium from BV-2 microglia stimulated by Nogo, Aβ production and tau phosphorylation in cultured neurons were increased. The conditioned medium also increased the expression of APP, its amyloidogenic processing, and the activity of GSK3β in neurons. The conditioned medium was also proinflammatory medium, and the blockage of the Nogo/NgR pathway improved the neuroinflammatory environment in APP/PS1 mice.

**Conclusions:**

Taken together, the neuroinflammation mediated by Nogo/NgR pathway in microglia could directly take part in the pathological process of AD by influencing the amyloidogenesis and tau phosphorylation. These results contribute to a better understanding of AD pathogenesis and could offer a new therapeutic option for delaying the progression of AD.

**Electronic supplementary material:**

The online version of this article (doi:10.1186/s12974-016-0522-x) contains supplementary material, which is available to authorized users.

## Background

Alzheimer’s disease (AD) is the most frequent cause of dementia [1]. More than 24 million people are affected in the world [2]. The two core pathological hallmarks of AD are β-amyloid (Aβ) plaques and neurofibrillary tangles (NFTs).

Nogo-A is a myelin-associated inhibitory molecule that inhibits the growth of neurite and reconnection in CNS diseases through cellar and molecular events [3, 4]. Nogo receptor (NgR) is a glycosylphosphatidylinositol-linked receptor, which binds with high affinity to Nogo-66, a hydrophilic 66 amino acid-long region of Nogo-A [5]. Recently, a number of studies have suggested that Nogo and its receptor participate in the AD pathogenesis. For example, it has been demonstrated that the expression of Nogo-A is increased in the hippocampus of patients with AD and is also localized in senile plaques around amyloid deposits [6]. Nogo-A shifts to the neuronal perikarya in human AD brain and amyloid precursor protein (APP)/PS1 transgenic mice [7]. Moreover, deleting Nogo ameliorates learning and memory deficits of APP transgenic mice [8]. Furthermore, the expression of NgR and downstream signaling molecules are increased in patients with AD and aged rats with deficits of spatial cognition [9, 10], and NgR also diffuses around amyloid plaques [7]. Recently, a study has shown that neuronal overexpression of NgR impairs cognitive function in AD transgenic mice [11]. Intracerebroventricular and subcutaneous injection of NgR(310)ecto-Fc reduce Aβ plaque load in the brain [7, 12] and subcutaneous infusion improves spatial memory in APPswe/PSEN-1ΔE9 transgenic mice [12]. Thus, despite several studies that imply the relationship between the Nogo, NgR, and AD, only a few detailed mechanisms have been provided to date.

Neuroinflammation has been recognized as an important cause of the AD and parallels disease severity [13, 14]. Many researchers have found that microglia, primary immune cells of the brain, play a central role in the pathogenesis of AD. Microglia are key innate immune cells that mediate inflammatory process in the AD brain [15]. In AD mouse models and patients, microglia are activated and recruited to Aβ deposits [16, 17]. Our previous research has confirmed that NgR is also expressed on microglia and the binding of Nogo with NgR could inhibit the microglia adhesion and migration through RhoA pathway in vitro [18]. The interaction of Nogo peptide with NgR expressed on microglia elevates the expression of proinflammatory enzymes and cytokines in vitro, which is mediated by the nuclear factor-kappa B (NF-κB) and signal transducers and activators of transcription3 (STAT3) pathways [19]. These findings initiate an interesting speculation about whether neuroinflammatory environment produced by Nogo-stimulated microglia play a role in the pathogenesis of AD. In this study, we explored whether the Nogo/NgR signaling pathway in the microglia participate in the AD pathogenesis. The results showed that the blockage of Nogo/NgR signaling pathway ameliorated AD pathological features including Aβ deposition and tau hyper-phosphorylation in APP/PS1 mice. Moreover, their mechanism may be related to the neuroinflammatory environment induced by Nogo/NgR signaling pathway in microglia.

## Methods

### Animals

C57BL/6J mice were obtained from Zhejiang Laboratory Animal Center (Hangzhou). APP/PS1 transgenic mice were purchased from the animal model center of Nanjing University (Nanjing, China); the mice were generated from the B6C3-Tg (APPswe, PSEN1dE9) 85Dbo/J double transgenic mouse line (Stock # 004462) provided by the National Jackson Animal Center (Bar Harbor, Maine, USA). All of the mice were raised in a thermostatic 12-h/12-h dark-light cycle environment, with free access to food and water. All animal tests were carried out in accordance with the US National Institute of Health (NIH) Guide for the Care and Use of Laboratory. All experimental procedures were approved by Institutional Animal Care and Use Committee (IACUC) of the Nanjing Medical University Experimental Animal Department.

### NEP1-40 treatment

To administer NEP1-40 peptide to mice, APP/PS1 mice at 6 months were anesthetized using chloral hydrate (100 mg/kg, i.p.), and a burr hole was drilled on the skull. A cannula (Alzet brain infusion kit II; Alza, Palo Alto, CA) was stereotactically introduced into the right lateral ventricle at the coordinates: 0.6 mm posterior and 1.2 mm lateral to the bregma and 2.0 mm deep to the pial surface. The cannula was held in place with cyanoacrylate, and the catheter was attached to osmotic micropump (Alzet 2004, 0.25 μl/h for 28 d; Alza). The pump was placed subcutaneously in the midscapular area of the back of the mice. Animals were categorized into vehicle (97.5 % PBS + 2.5 % DMSO)-treated and NEP1-40 (500 μM in vehicle)-treated groups. Pumps were replaced after 28 days and connected to the same cannula.

### Tissue preparation

After infusion for 2 months, mice were deeply anesthetized using chloral hydrate (100 mg/kg, i.p.) and perfused intracardially with cold 4 % paraformaldehyde (PFA) in PBS followed by perfusion with PBS. Brain tissues were fixed with PFA overnight at 4 °C and equilibrated by immersing in 15 and 30 % sucrose in PBS overnight at 4 °C, respectively. The brain tissues were then sectioned to a thickness of 15 μm on a Leica-1900 cryostat (Leica Instruments, Germany). The sections were used for immunohistochemistry or thioflavin S staining and stored at −80 °C.

### Immunohistochemistry

Immunohistochemistry was performed as described earlier with some modifications [20]. Briefly, the brain sections were degreased in acetone for 20 min at 4 °C. Then, the sections were incubated with primary antibodies overnight at 4 °C after incubation with a blocking solution (10 % normal goat serum, 0.3 % Triton X-100 in PBS) at room temperature for 1 h. The following primary antibodies were used: rabbit anti-Nogo-A polyclonal antibody (1:50; Santa Cruz Biotechnology, Santa Cruz, CA, USA), mouse anti-6E10 monoclonal antibody (1:500; Covance, Princeton, NJ), and rabbit anti-Iba-1 polyclonal antibody (1:200; Wako Chemicals, Japan). After rinsing in PBS, the sections were incubated with secondary antibodies: Alexa Fluo-488 conjugated goat anti-rabbit IgG antibody (1:300; Invitrogen), Alexa Fluo-594 conjugated goat anti-mouse IgG antibody (1:500; Invitrogen), and Hoechst 33342 to counterstain nuclei when necessary.

In thioflavin S (ThioS, Sigma) staining [21], the brain sections were incubated with a 1 % ThioS solution dissolved in distilled water containing 50 % ethanol for 5 min and differentiated in 50 % ethanol thrice. The fluorescent imaging was visualized by using Olympus IX-81 inverted fluorescence microscope and FV1000 confocal laser scanning microscope (Olympus Corporation, Japan). For quantification of amyloid load, the area of the five sections of the cortex and hippocampus in each group was calculated and analyzed using the Image-Pro Plus software.

### ELISA

For Aβ ELISA assay, the brain was homogenized as described previously [22]. Briefly, cortical and hippocampal tissues were homogenized in TBS containing a protease inhibitor cocktail (Roche) and then centrifuged at 16,000*g* for 30 min at 4 °C. The supernatant (TBS-soluble fraction) was collected and stored at −80 °C. The pellets were homogenized in TBS plus 1 % Triton X-100 (TBS-T) containing a protease inhibitor cocktail (Roche), sonicated for 5 min at 4 °C in a water bath, and centrifuged at 16,000*g* for another 30 min at 4 °C. The supernatant (TBS-T-soluble fraction) was collected and stored at −80 °C. The pellets were extracted for a third time with an ice-cold guanidine buffer (5 M guanidine HCl/50 mM Tris, pH 8.0) and in hence referred to as the guanidine-soluble fraction. The protein concentration of all samples was measured using a bicinchoninic acid protein assay kit (Beyotime Biotechnology). The concentrations of Aβ in three separate fractions of brain samples were determined using Aβ42 and Aβ40 ELISA kits (Invitrogen) following the manufacturer’s instructions.

Brain tissues were homogenized in cell lysate buffer (RayBiotech. Inc., San Diego, CA) supplemented with a protease inhibitor cocktail (Roche) and centrifuged at 12,000*g* for 20 min at 4 °C. The supernatant was collected and stored at −80 °C. The protein concentration was measured using a bicinchoninic acid protein assay kit (Beyotime Biotechnology). The proportions of interleukin-1β (IL-1β) and interleukin-4 (IL-4) were examined using IL-1β and IL-4 ELISA kits (RayBiotech. Inc.) following the manufacturer’s instructions.

### Western blot analysis

After 2 months of administration, mice were deeply anesthetized with chloral hydrate (100 mg/kg, i.p.). After perfusion with PBS, the brain was quickly dissected and stored at −80 °C until further use. Snap-frozen brain tissue was homogenized in RIPA buffer (Beyotime Biotechnology) supplemented with a protease inhibitor cocktail (Roche). Extracts were centrifuged at 12,000*g* for 20 min at 4 °C, and the supernatant was collected and the protein concentration was determined using a bicinchoninic acid protein assay kit (Beyotime Biotechnology).

Neurons obtained from different treatments were lysed in RIPA buffer (Beyotime Biotechnology) containing a protease inhibitor cocktail (Roche). The cell extracts were centrifuged at 12,000*g* at 4 °C for 20 min to remove cell debris. The supernatant was collected and the protein concentration was determined using a bicinchoninic acid protein assay kit (Beyotime Biotechnology, China).

Supernatant protein (50 μg) was electrophoretically separated using denaturing gels and transferred onto nitrocellulose membranes. Membranes were blocked for 1 h at room temperature with 5 % bovine serum albumin in Tris-buffered saline Tween-20 and then incubated overnight at 4 °C with specific primary antibody. The following antibodies were used: mouse anti-APP polyclonal antibody (1:500; Sigma), mouse anti-Presenilin-1 polyclonal antibody (1:500; Millipore), rabbit anti-BACE1 polyclonal antibody (1:800; Millipore), mouse anti-β-CTF polyclonal antibody (1:1000, Sigma), rabbit anti-a disintegrin and metalloproteinases 10 (ADAM10) polyclonal antibody (1:800; Millipore), rabbit anti-tau-1 polyclonal antibody (1:500; Millipore), rabbit anti-p-tau at Thr202/205 polyclonal antibody (1:500; Santa Cruz Biotechnology), rabbit anti-p-tau at Ser396 polyclonal antibody (paired helical filament (PHF) 13, 1:1000; Cell Signaling Technology Inc., Beverley, MA, USA), rabbit anti-GSK-3β polyclonal antibody (1:1000; Cell Signaling Technology Inc.), rabbit anti-p-GSK3β at pY216 polyclonal antibody (1:1000; Abcam, Cambridge, MA, USA), rabbit anti-inducible nitric oxide synthase (iNOS) polyclonal antibody (1:800; Abcam), goat anti-cyclooxygenase-2 (COX-2) polyclonal antibody (1:500; Santa Cruz Biotechnology Inc.), and mouse anti-β-actin monoclonal antibody (1:2000; Santa Cruz Biotechnology). After immunoblotting with horseradish peroxidase-conjugated secondary antibodies goat anti-mouse IgG (1:10000; Sigma), rabbit anti-goat IgG (1:500; R&D System, Minneapolis, MN, USA), or goat anti-rabbit IgG (1:5000; Cell Signaling Technology Inc.) conjugated with horseradish peroxidase, immunoreactive bands were detected by chemiluminescence reagents (ECL; Millipore).

The images of protein bands were captured with a Bio-Rad Gel Doc XR documentation system for blot densitometry assay. The relative expressions of the protein were determined by scanning the pixel density of resultant blots using Quantity One software.

### Quantitative real-time PCR (RT-PCR)

For RT-PCR, total RNA was extracted from the brain tissue or BV-2 microglia cells in TRIzol on ice (Vazyme, NJ, China). HiScript First Strand cDNA Synthesis Kit (Vazyme) was used to convert RNA to cDNA. To quantify RNA, real-time PCR was performed using AceQTM qPCR SYBR Green Master Mix (Vazyme) on a LightCycler96 PCR system (Roche). The cycle time values of each sample were normalized to GAPDH.

The following PCR primer sequences were used for detecting transcriptions: GAPDH F: 5′-GGTGAAGGTCGGTGTGAACG-3′, R: 5′-CTCGCTCCTGGAAGATGGTG-3′; iNOS F: 5′-ACCTTGTTCAGCTACGCCTT-3′, R: 5′-CATTCCCAAATGTGCTTGTC-3′; IL-1β F: 5′-TCAGGCAGGCAGTATCACTC-3′, R: 5′-CATGAGTCACAGAGGATGGG-3′; tumor necrosis factor-α (TNF-α) F: 5′-TCTCTTCAAGGGACAAGGCT-3′, R: 5′-GGCAGAGAGGAGGTTGACTT-3′; IL-6 F: 5′-ACTTCACAAGTCGGAGGCTT-3′, R: 5′-TTGCCATTGCACAACTCTTT-3′; COX-2 F: 5′-ATGAGCACAGGATTTGACCA-3′, R: 5′-TGGGCTTCAGCAGTAATTTG-3′; chemokine (C-C motif) ligand 2 (CCL2) F: 5′-GCATCTGCCCTAAGGTCTTC-3′, R: 5′-AAGTGCTTGAGGTGGTTGTG-3′; arginase 1 (Arg1) F: 5′-CAGTGGCTTTAACCTTGGCT-3′, R: 5′-GTCAGTCCCTGGCTTATGGT-3′; found in inflammatory zone 1 (Fizz1) F: 5′-CTGCTACTGGGTGTGCTTGT-3′, R: 5′-GGCAGTTGCAAGTATCTCCA-3′; chitinase-like 3 (Chil3, Ym1) F: 5′-TCTATGCCTTTGCTGGAATG-3′, R: 5′-CAGGTCCAAACTTCCATCCT-3′; IL-4 F: 5′-TGTACCAGGAGCCATATCCA-3′, R: 5′-TTCTTCGTTGCTGTGAGGAC-3′; cd206 F: 5′-TGATTACGAGCAGTGGAAGC-3′, R: 5′- GTTCACCGTAAGCCCAATTT-3′; interleukin-10 (IL-10) F: 5′-CAGAGCCACATGCTCCTAGA-3′, R: 5′-GGCAACCCAAGTAACCCTTA-3′. The primers were synthesized by Nanjing Genscript (Nanjing, China).

### Cell cultures and treatments

BV-2 murine microglia cell was routinely grown in Dulbecco’s modified Eagle’s medium (DMEM, Gibco, Carlsbad, CA, USA) supplemented with 10 % fetal bovine serum (FBS, Gibco), penicillin (0.1 %), and streptomycin (0.1 %) at 5 % CO_2_, 37 °C.

BV-2 microglia cells were treated according to the method described with modifications [23]. Briefly, 96-well plates (Corning, New York, USA) were coated with methanol-solubilized nitrocellulose and washed with ddH_2_O. Then, the wells were incubated with poly-L-lysine (PLL, 0.05 mg/ml, Sigma, St Louis, MO, USA) for 2 h at 37 °C and washed with ddH_2_O. Later, Nogo-P4 (Alpha Diagnostic International Inc., San Antonio, TX) was applied at 100 μg/mL and coated overnight at 4 °C. Nogo-P4 is a 25 aa inhibitory peptide sequence (residues 31–55 of Nogo-66), a potent inhibitory component of Nogo-A [3]. After transfection, BV-2 microglia cells were added onto the wells pre-coated with Nogo-P4 or PBS at a density of 5 × 10^5^ cells/mL and cultured for 6 h. Then, conditioned medium was collected and centrifuged to discard cell debris for further experiment.

Primary cortical neuron cultures were prepared following the described method with some modifications [24]. Briefly, cortices of C57BL/6J mouse at embryonic 15–17 days were removed, dissected free of meninges, and dissociated in 0.125 % trypsin. Then, dissociated cells were plated in PLL (0.05 mg/ml, Sigma)-coated 24- or 96-well plates at a density of 3 × 10^5^ cells/mL in DMEM supplemented with 10 % FBS and cultured at 37 °C in 5 % CO_2_. After 4 h, the medium was replaced with neurobasal medium (Gibco) supplemented with B27 supplements (Gibco). About 95 % of these cells were positive for microtubule-associated protein 2 (MAP2), a marker for neurons. After 6 days of in vitro culture, the medium was replaced with BV-2 microglia-conditioned medium for a further 24 h.

### Transfection

According to the gene sequence of mouse NgR, a small interfering RNA (siRNA) targeting NgR (NgR siRNA, sense: 5′-UUCUCCGAACGUGUCACGUTT-3′ and antisense: 5′-ACGUGACACGUUCGGAGAATT-3′) was designed. Non-specific sequences (control siRNA, sense: 5′-GCCGAAAUCUCACUAUCCUTT-3′ and antisense: 5′-AGGAUAGUGAGAUUUCGGCTT-3′) were used as a control. The siRNA was synthesized by Shanghai GenePharma Co. Ltd. (Shanghai, China).

BV-2 microglia cells were seeded onto 24-well plates at the density of 1 × 10^5^ cells/well and cultured overnight. Before transfection, the medium was changed to a medium containing no serum for 2 h. Lipofectamine® 2000 Transfection Reagent (Invitrogen, Carlsbad, CA, USA) was used to transfect the siRNAs. Treatment with control siRNA or NgR siRNA was realized at a final dose of 60 nM siRNA/well and replaced to a fresh medium after 5 h. These cells were subsequently cultured for 31 h.

### Quantification of Aβ40 and Aβ42 levels

Primary cortical neurons were treated with BV-2 microglia-conditioned medium for 24 h, and the secretion and production of Aβ were detected as described previously [25, 26]. The medium of neurons was then collected and centrifuged for 20 min at 12,000*g* to remove cell debris, and then, the levels of secreted Aβ40 and Aβ42 were measured using mouse Aβ40 ELISA kits (Invitrogen) and mouse Aβ42 ELISA kits (Invitrogen) following the manufacturer’s instructions. To determine the production of intracellular Aβ, the treated cells were lysed with cold radio immune precipitation assay buffer (Roche) containing a protease inhibitor cocktail (Roche) and centrifuged at 12,000*g* for 20 min to remove cell debris. The proportions of Aβ40 and Aβ42 in protein extracts were measured using mouse Aβ40 ELISA kits (Invitrogen) and mouse Aβ42 ELISA kits (Invitrogen) according to the manufacturer’s instructions. The levels of Aβ were normalized to the amount of total protein in neurons.

### Measurement of secretase activity

Primary cortical neurons were harvested, and the activity of secretases was measured, as described previously with some modifications [27]. Briefly, the cells were lysed in extraction buffer on ice for 10 min, centrifuged at 10,000*g* at 4 °C for 5 min, and the protein concentration was quantified. β-secretase activity was measured using β-secretase activity kit (Biovision, Milpitas, CA, USA) according to the manufacturer’s instructions. Fluorescence was measured with an emission and excitation wavelength of 495–510 and 335–355 nm, respectively, using a Safire2 microplate reader (TECAN, Salzburg, Austria). The activity of α-secretase was measured using the fluorogenic α-secretase substrate II with EDANS/DABCYL (480 nm emission and 330 nm excitation, Millipore), and γ-secretase activity was measured using a fluorogenic γ-secretase substrate with NMA/DNP (435 nm emission and 330 nm excitation, Millipore) 12 h after incubation in the same lysate.

### Thioflavin-T (ThT) binding assay

After stimulated by PBS or Nogo-P4, the effects of BV-2 microglia-conditioned medium on Aβ aggregation and fibrillar Aβ depolymerization were examined using ThT (Sigma) binding assay [28, 29]. Specifically, Aβ_1−42_ (AnaSpec Inc., San Jose, CA) was prepared as a 1-mg/mL stock solution in DMSO (Sigma). ThT solution was prepared as a 1-mg/mL stock solution in ddH_2_O and used at a final concentration of 5 mM in PBS (pH 7.4). Aβ_1–42_ monomers were prepared at the final desired concentration (10 μM) in PBS (pH 7.4) and were added to the appropriate wells of a 96-well black plate (10 μl/well). An equal volume of the conditioned medium or PBS solution was added to the Aβ_1–42_-containing wells. Plates were then placed in 37 °C. After 24 h, 180-μl ThT solution was added to the wells. Following 1-min incubation at room temperature, Aβ peptide aggregation was measured (Safire2 microplate reader, TECAN) as the increase in relative ThT fluorescence (excitation at 450 nm, emission at 480 nm). To test the effects of the conditioned medium on fibrillar Aβ_1–42_ depolymerization, 20-μl Aβ_1–42_ monomers (20 μM) were added to a 96-well black plate. After incubation for 24 h at 37 °C, an equal volume of the conditioned medium or PBS solution was added to the Aβ_1–42_-containing wells and 160-μl ThT solution was added to each well. Following 1-min incubation at room temperature, ThT fluorescence was measured. Effects of microglia-conditioned medium on Aβ_1–42_ aggregation and depolymerization were represented as relative changes in ThT fluorescence as compared to fluorescence of the Aβ_1–42_ solution.

### Statistical analysis

All presented data represent the results of three independent experiments. Statistical analysis was performed using Student’s *t* test or one-way analysis of variance (ANOVA) with post hoc Tukey’s test using Graph Pad Prism 5 software (Graph Pad Software Inc., La Jolla, CA, USA). All data were presented as mean ± SD. *p* value of *p < 0.05 was considered statistically significant.

## Results

### Inhibition of the Nogo/NgR pathway reduced Aβ plaque deposition in APP/PS1 mice

To investigate the effects of Nogo/NgR pathway on pathological features of AD, NEP1-40 was intracerebroventricularly administered by continuous infusion into APP/PS1 mice aged from 6 to 8 months old with an Alzet mini-pump. APP/PS1 transgenic mice is a commonly used AD animal model [30, 31]. NEP1-40 is a competitive antagonist peptide of Nogo/NgR pathway derived from the first 40 amino acids of the Nogo-66, prevents the combination of Nogo with NgR, and enables the promotion of the axonal outgrowth through blocking the binding of Nogo to NgR in vitro and in vivo [32–35].

**Fig. 1:**
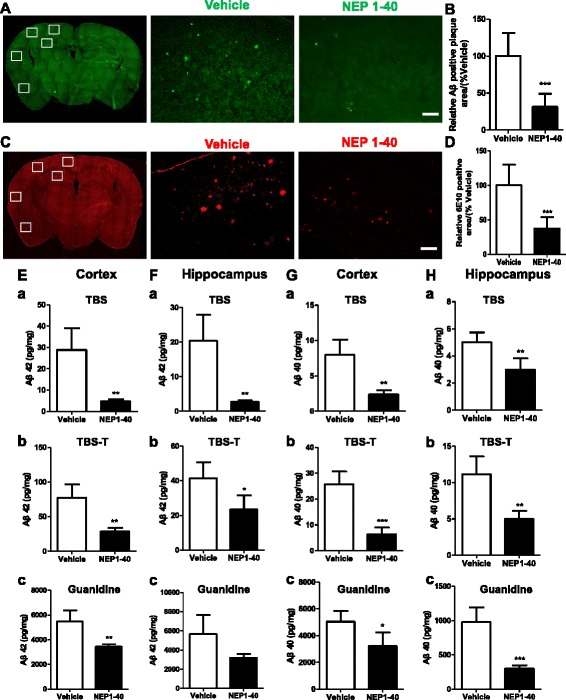
Inhibition of the Nogo/NgR pathway reduced Aβ plaque deposition in APP/PS1 mice. **a, b** Mice brain sections from APP/PS1 mice were stained with thioflavin S and calculated as Aβ-positive area fraction in the hippocampal and cortical regions at 8 months of age. Bar = 100 μM. Values were reported as mean ± SD, as a percentage of values determined in the vehicle group (control, 100 %). The Aβ plaque in the brain was estimated after immunohistochemistry with Aβ antibody (6E10), and the position area fraction of the hippocampal and cortical regions of brain tissues were calculated (**c, d**). Bar = 100 μM. Values were reported as mean ± SD, as a percentage of values determined in the vehicle group (control, 100 %). **e–h** The cortex or hippocampus of APP/PS1 mice was homogenized and separated into TBS-, TBST-, and guanidine-soluble fractions. The proportions of Aβ42 (**e, f**) and Aβ40 (**g, h**) were measured using ELISA. **e**, **g** The cortex of APP/PS1 mice; **f**, **h** The hippocampus of APP/PS1 mice. Values were reported as mean ± SD. *p < 0.05; **p < 0.01; ***p < 0.001, when compared with the vehicle group, *n* = 3–6

After 2 months, mice were killed, and in order to determine the deposition of Aβ plaque in APP/PS1 mice, the brain sections were stained for fibrillar Aβ with ThioS or 6E10 (Aβ antibody). Quantitative analysis in the hippocampal and cortical regions revealed significant reduction in the deposition of Aβ plaque in APP/PS1 mice after NEP1-40 treatment as compared with the vehicle group (Fig. 1a–d). To further validate the immunohistochemical results, the levels of different Aβ isoforms by performing ELISA of cortical and hippocampal homogenates derived from the vehicle- or NEP1-40-infused APP/PS1 mice were measured. As shown in Fig. 1e–h, NEP1-40 administration significantly decreased the concentrations of Aβ42 and Aβ40 in TBS-soluble fractions, TBS-T-soluble fractions, and guanidine chloride-soluble fractions, which were enriched in monomeric, oligomer, and high molecular weight aggregated Aβ species in the cortex and hippocampus. These results imply that inhibition of Nogo/NgR pathway could attenuate amyloid plaque deposition in AD animal model.

### Blockage of the Nogo/NgR pathway attenuated amyloidogenic and induced non-amyloidogenic processing of APP in APP/PS1 mice

**Fig. 2:**
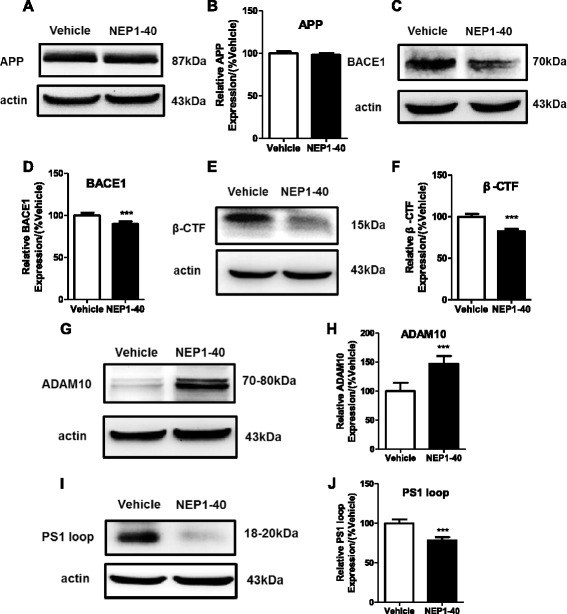
Blockage of Nogo/NgR pathway attenuated amyloidogenic and induced non-amyloidogenic processing of APP in APP/PS1 mice. Homogenates from the brain tissue of 8-month-old APP/PS1 transgenic mice were subjected to western blot analysis with markers of APP expression and processing. **a**, **b** APP. **c, d** BACE1. **e, f** β-CTF. **g, h** ADAM10. **i, j** PS1 loop. Values were reported as mean ± SD, as a percentage of values determined in the vehicle group (control, 100 %). ***p < 0.001, when compared with the vehicle group, *n* = 3–6

Aβ is a cleavage product of APP. APP protein can be processed by α-secretase in a non-amyloidogenic pathway and also can be processed by β- and γ-secretases in an amyloidogenic pathway which produces Aβ [36]. In order to study the expression and processing of APP, the expression of APP and secretases in APP/PS1 mice was examined using western blot. Results in Fig. 2a, b showed that there was no difference between the vehicle and the NEP1-40 group, indicating that basal level of APP expression was unaltered after infusion of NEP1-40 in APP/PS1 mice. However, NEP1-40 treatment markedly reduced BACE1 (Fig. 2c, d) and β-CTF (Fig. 2e, f) levels, suggesting that the expression and processing of β-secretase were decreased by blocking the Nogo/NgR pathway. Moreover, the expression of α-secretase (ADAM10) was increased by NEP1-40 treatment in APP/PS1 mice (Fig. 2g, h). Next, we noticed that the expression of PS1 loop (a key component of γ-secretase complex) was decreased in the NEP1-40 group compared with the vehicle group (Fig. 2i, j). These data suggest that NEP1-40 treatment, blocking the Nogo/NgR pathway, induced non-amyloidogenic processing of APP and inhibited amyloidogenic processing of APP. Hence, the observed reduction in amyloid accumulation might be attributable to the regulation of metabolic processing of APP.

### Decreasing of the phosphorylated levels of tau in APP/PS1 mice after inhibition of the Nogo/NgR pathway

**Fig. 3:**
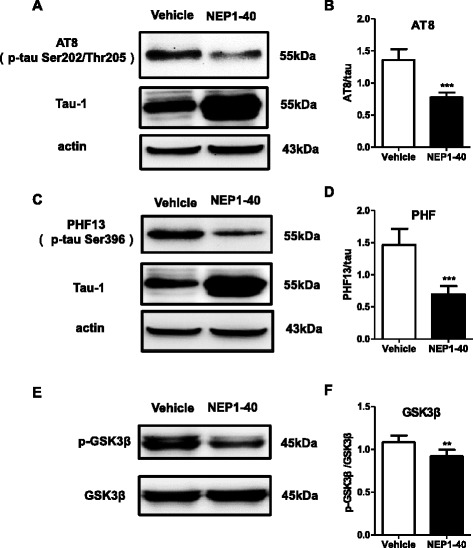
Decreasing the phosphorylated levels of tau in APP/PS1 mice after inhibition of the Nogo/NgR pathway. The phosphorylation of tau (**a–d**) and activation of GSK-3β (**e, f**) in the brain homogenates was quantified with western blot. **a**, **b** AT-8. **c, d** PHF13. Values were reported as mean ± SD. *p < 0.05; **p < 0.01, when compared with the vehicle group, *n* = 3–6

Another key pathological characteristic of AD is NFTs which are composed predominantly of hyperphosphorylated tau protein and assembled primarily in the PHF conformation [37]. Thus, we next explored the effect of inhibition of the Nogo/NgR pathway on the phosphorylation of tau using western blot. After NEP1-40 infusion, the phosphorylated levels at Ser202/Thr205 of tau detected by AT8 (Fig. 3a, b) and at Ser396 detected by PHF13 were decreased in the APP/PS1 mice (Fig. 3c, d). Anti-tau-1 antibody recognizes a non-phosphorylated tau epitope, the higher expression of tau-1 reflected the reduction of phosphorylated levels of tau. Moreover, significant reduction in phosphorylated GSK3β, an important tau kinase [38], was found in NEP1-40-treated mice (Fig. 3e, f). These results indicate that the inhibition of the Nogo/NgR pathway could alter tau phosphorylation through inhibiting the activity of GSK3β and thus potentially affect the accumulation of NFTs in the AD mice brain.

### The conditioned medium (CM) from BV-2 microglia activated by the Nogo/NgR pathway increased the production and the aggregation of Aβ in primary neurons

**Fig. 4:**
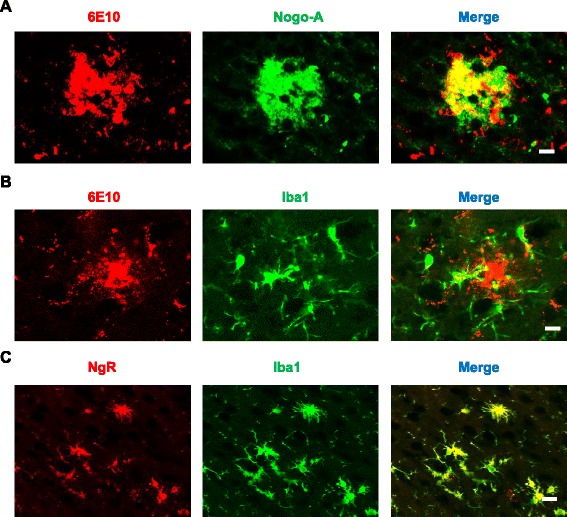
Nogo-A and NgR localization in APP/PS1 mice brain. **a** Mice brain sections were processed for anti-Aβ (6E10) and anti-Nogo-A immunohistology as indicated. Bar = 50 μM. **b** Mice brain sections were processed for anti-Aβ (6E10) and anti-Iba1 immunohistology as indicated. Bar = 50 μM. **c** Mice brain sections were processed for anti-NgR and anti-Iba1 immunohistology as indicated. Bar = 50 μM

Above results showed that the Nogo/NgR signaling pathway was involved in the development of AD pathological features in APP/PS1 mice, but the mechanism remained unclear. It has been reported that Nogo-A is localized in senile plaques in patients with AD [6]. Consistent with the results, we found that Nogo-A was co-localized with Aβ plaques stained by 6E10 in APP/PS1 transgenic mice (Fig. 4a). Moreover, microglia was also co-localized with Aβ plaques (Fig. 4b) and NgR was expressed on the Iba^+^ microglia (Fig. 4c). Our previous study found that Nogo/NgR signaling pathway is involved in neuroinflammation through increasing the expression of inflammatory mediators in microglia in vitro [19]. Hence, these data suggest that neuroinflammatory environment induced by the Nogo/NgR pathway might increase the production and the aggregation of Aβ in primary neurons.

**Fig. 5:**
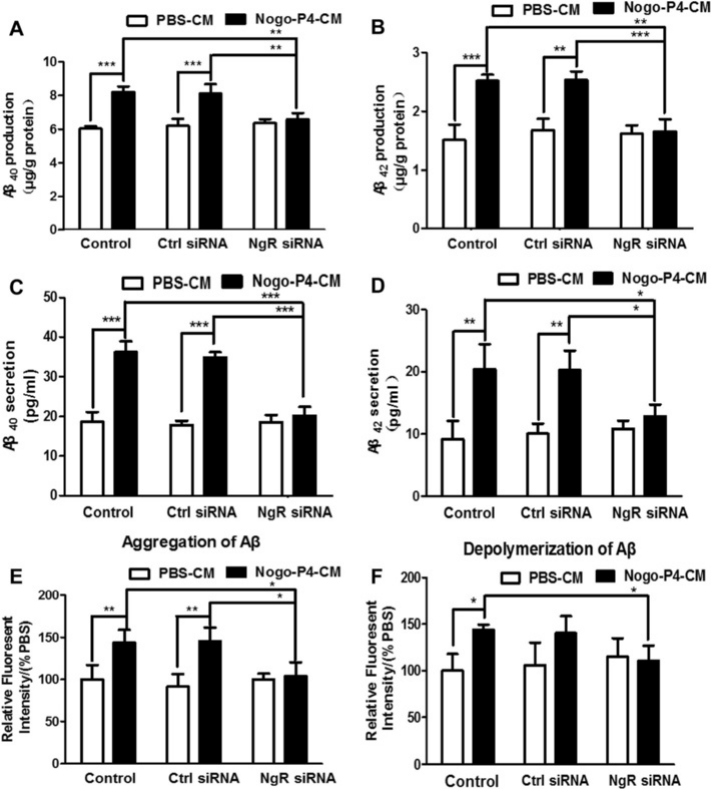
CM from BV-2 microglia activated by Nogo/NgR pathway increased the production and aggregation of Aβ. After exposing for CM of BV-2 microglia for 24 h, ELISA was performed to detect the production of Aβ40 (**a**) and Aβ42 (**b**) and the secretion of Aβ40 (**c**) and Aβ42 (**d**). Values were reported as mean ± SD. The aggregation of Aβ (**e**) and the depolymerization of fibrillar Aβ (**f**) were determined using ThT binding assay. Values were reported as mean ± SD, as a percentage of values determined in PBS group (control, 100 %). *p < 0.05, **p < 0.01, ***p < 0.01, *n* = 3

To investigate the hypothesis, NgR in BV-2 microglia was silenced by siRNA interference and then primary neurons were exposed to the CM prepared from Nogo-P4 stimulated BV-2 microglia for 24 h. The expression of NgR in BV-2 microglia was significantly decreased by NgR siRNA interference (Additional file 1: Figure S1A, B), and the cellular ability was not affected (Additional file 1: Figure S1C). The levels of Aβ40 (Fig. 5a) and Aβ42 (Fig. 5b) in cell lysates and in the medium (Aβ40, Fig. 5c; Aβ42, Fig. 5d) in neurons exposed to the CM were significantly increased compared with neurons exposed to the control PBS medium. Compared with control siRNA-treated CM, NgR siRNA treatment significantly attenuated the production of Aβ40 (Fig. 5a) and Aβ42 (Fig. 5b) in cell lysates and in the medium (Aβ40, Fig. 5c; Aβ42, Fig. 5d) when neurons were induced by Nogo-P4. The background secretion of Aβ in CM was eliminated (Additional file 2: Figure S2). Furthermore, the effects of the CM on the aggregation and depolymerization of Aβ_1–42_ was investigated. Results in Fig. 5e, f showed that the CM promoted the aggregation of Aβ_1–42_ and inhibited depolymerization of Aβ_1–42_, which was reversed by the treatment with NgR siRNA. Results above indicated that the CM generated from Nogo/NgR-activated BV-2 microglia promoted the production and release of Aβ in neurons and affected the aggregation and depolymerization of Aβ_1–42_.

### The CM derived from Nogo/NgR-activated BV-2 microglia increased the APP expression and accelerated the amyloidogenic processing of APP in primary neurons

**Fig. 6:**
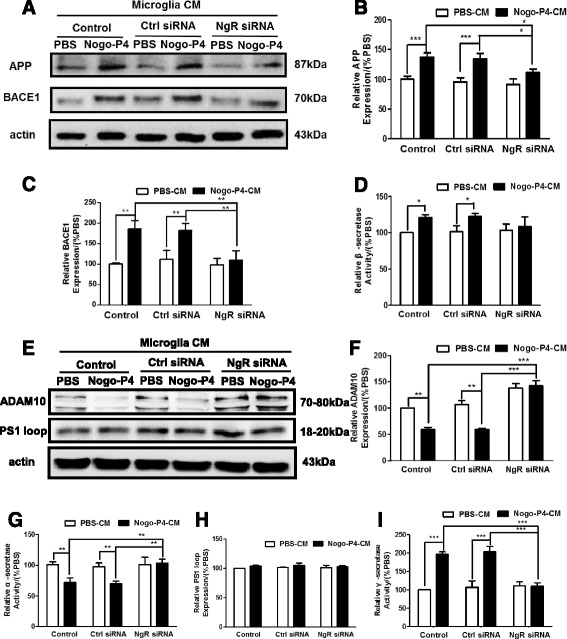
The CM derived from Nogo/NgR-activated BV-2 microglia increased the expression and amyloidogenic processing of APP. Neurons were exposed for CM of BV-2 microglia at different treatments for 24 h. The expression of APP (**a, b**), BACE1 (**a, c**), ADAM10 (**e, f**), and PS1 loop (**e, h**) was determined using western blot. Values were reported as mean ± SD, as a percentage of values determined in the PBS group (control, 100 %). FRET assay was performed to detect the activity of β-secretase (**d**), α-secretase (**g**), and γ-secretase (**i**). Values were reported as mean ± SD, as a percentage of values determined in the PBS group (control, 100 %). *p < 0.05, **p < 0.01, ***p < 0.01, *n* = 3

The effects of the CM derived from Nogo/NgR-activated BV-2 microglia on the expression and the processing of APP were determined using western blot. As shown in Fig. 6a, b, after neurons were stimulated by CM derived from Nogo-P4-activated BV-2 microglia, the expression of APP was increased in neurons and NgR siRNA inhibited the effect of Nogo-P4. Moreover, it was measured whether the processing of APP was affected by the CM. When neurons were treated with the CM, a significant increase in the expression of BACE1 and activity of β-secretase in neurons was observed (Fig. 6a, c, d). The expression of ADAM10 and activity of α-secretase were significantly decreased in neurons treated with the CM (Fig. 6e–g). Although the expression of PS1 loop (a key component of γ-secretase complex) was unaffected (Fig. 6e, h), the activity of γ-secretase was induced by the CM (Fig. 6i). Furthermore, NgR siRNA treatment significantly attenuated the effects of Nogo-P4 on the expression and activity of secretase compared with control siRNA. Results above suggested that the CM from BV-2 microglia activated by Nogo/NgR is responsible for the expression and amyloidogenic processing of APP in neurons.

### Tau phosphorylation level in primary neurons were promoted by the CM from Nogo/NgR-activated BV-2 microglia

**Fig. 7:**
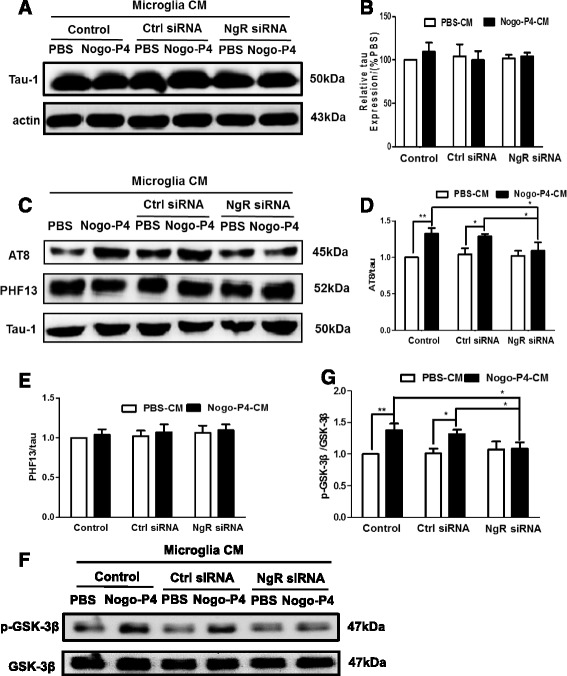
Tau phosphorylation in primary neurons were promoted by the CM from Nogo/NgR-activated BV-2 microglia. Neurons were exposed to the CM of BV-2 microglia at different stimulations for 24 h. The expression of tau (**a, b**) was determined using western blot. Values were reported as mean ± SD, as a percentage of values determined in the PBS group (control, 100 %). Effects of CM from Nogo/NgR pathway activated BV-2 microglia on tau phosphorylation (**c–e**) and GSK-3β activation (**f, g**) in primary neurons were measured using western blot. Values were reported as mean ± SD. *p < 0.05, **p < 0.01, *n* = 3

Furthermore, we investigated whether the phosphorylated levels of tau were influenced by the CM from BV-2 microglia activated by Nogo/NgR. Results of western blot showed that there was no significant change in the expression of tau-1, the non-phosphorylated tau, among different conditions (Fig. 7a, b). After neurons were stimulated by the CM from Nogo-P4-activated BV-2 microglia for 24 h, the p-tau level at Ser202/Thr205 detected by AT8 in neurons was increased and the increase could be blocked by pretreatment with NgR siRNA (Fig. 7c, d). However, the CM did not alter the phosphorylated level of tau at Ser396 detected by PHF-13 (Fig. 7c, e). Moreover, after neurons were stimulated by the CM, the phosphorylated level of GSK-3β was enhanced, and the phosphorylation was attenuated by pretreatment with NgR siRNA (Fig. 7f, g). Therefore, the CM from the BV-2 microglia activated by Nogo/NgR contributes to the phosphorylation of tau and GSK-3β in primary cultured neurons.

### The expression of proinflammatory mediators was increased and anti-inflammatory mediators were decreased in the CM of BV-2 microglia activated by Nogo/NgR

**Fig. 8:**
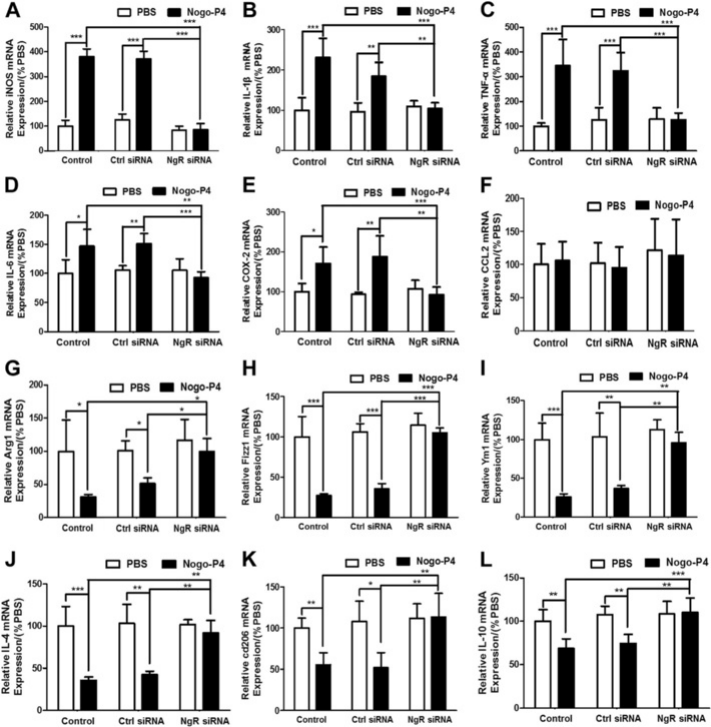
Increasing of M1 inflammatory gene and decreasing of M2 inflammatory gene in Nogo/NgR-activated BV-2 microglia. Before added to the protein-coated wells, BV-2 microglia was transfected with control or NgR siRNA to suppress the expression of NgR. The cells were then stimulated by exposed to PBS or Nogo-P4 for 6 h. The levels of M1 (**a–f**) and M2 (**g–l**) inflammatory gene transcripts in BV-2 microglia were examined by quantitative RT-PCR. **a** iNOS. **b** IL-1β. **c** TNF-α. **d** IL-6. **e** COX-2. **f** CCL2. **g** Arg1. **h** Fizz1. **i** Ym1. **j** IL-4. **k** cd206. **l** IL-10. Values were reported as mean ± SD, as a percentage of values determined in PBS group (control, 100 %). *p < 0.05, **p < 0.01, ***p < 0.01, *n* = 4

Our previous research found that Nogo-P4 elevated the expression of proinflammatory mediators in microglia in vitro, such as IL-1β, TNF-α, prostaglandin E2 (PGE2), nitric oxide (NO) [19]. In the present study we examined whether proinflammatory cytokines from microglia activated by the Nogo/NgR signaling pathway is involved in AD pathological features. As shown in Fig. 8, the levels of transcripts of M1-inflammatory gene markers (iNOS, IL-1β, TNF-α, IL-6, COX-2) from Nogo-P4 activated BV-2 microglia were higher than the control BV-2 microglia but the levels of the transcripts of M2-inflammatory gene markers (Arg1, Fizz1, Ym1, IL-4, cd206, IL-10) were lower. Moreover, NgR siRNA treatment significantly reversed the effects of Nogo-P4 on the expression of M1 and M2 inflammatory genes compared with control siRNA. These results implied that the Nogo/NgR pathway could induce the proinflammatory environment in BV-2 microglia, and the blockage of the Nogo/NgR pathway might change the neuroinflammatory environment from proinflammation to anti-inflammation.

**Fig. 9:**
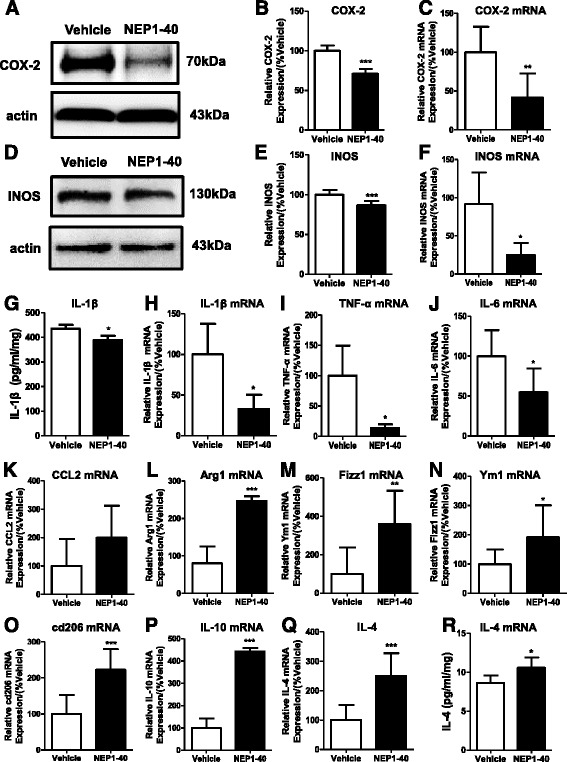
Inhibition of the Nogo/NgR pathway improved neuroinflammatory environment in APP/PS1 mice. Homogenates from brain tissue of 8-month-old APP/PS1 transgenic mice were subjected to western blot to measure the expression of COX-2 and iNOS. **a, b** COX-2. **d, e** iNOS. Values were reported as mean ± SD, as a percentage of values determined in the vehicle group (control, 100 %). The brain of APP/PS1 mice was homogenized, and the proportions of IL-1β (**g**) and IL-4 (**q**) were determined using ELISA. Values were reported as mean ± SD. Inflammatory gene transcripts in the brain from 8-month-old APP/PS1 mice were measured by quantitative RT-PCR. **c** COX-2. **f** iNOS. **h** IL-1β. **i** TNF-α. **j** IL-6. **k** CCL2. **l** Arg1. **m** Fizz1. **n** Ym1. **o** cd206. **p** IL-10. **r** IL-4. Values were reported as mean ± SD, as a percentage of values determined in the vehicle group (control, 100 %). *p < 0.05; **p < 0.01; ***p < 0.001, when compared with the vehicle group, *n* = 3–6

Furthermore, we investigated the neuroinflammation after the inhibition of the Nogo/NgR signaling pathway in APP/PS1 mice. The expression of inflammatory mediators in APP/PS1 mice was determined using western blot, ELISA and RT-PCR. Results showed that NEP1-40 administration decreased the levels of proinflammatory mediators including COX-2 (Fig. 9a–c), iNOS (Fig. 9d–f), IL-1β (Fig. 9g, h), TNF-α (Fig. 9i), and IL-6 (Fig. 9j), but no change in the transcription of the CCL2 was found (Fig. 9k). NEP1-40 infusion also promoted the expression of anti-inflammatory cytokines including Arg1 (Fig. 9l), Fizz1 (Fig. 9m), Ym1 (Fig. 9n), cd206 (Fig. 9o), IL-10 (Fig. 9p), and IL-4 (Fig. 5q–r) in the brain compared with the vehicle group. These results are consistent with those in vitro and indicate that the Nogo/NgR signaling pathway induced neuroinflammation in APP/PS1 transgenic mice, which might contribute to the production of the pathological features in AD.

## Discussion

In this study, the evidence of an important regulation of the formation of Aβ plaques and hyperphosphorylation of tau by neuroinflammation triggered by Nogo/NgR pathway in vivo and in vitro is presented. From the results presented above, two points are particularly noteworthy. Firstly, the results from the present study in vivo confirmed that Nogo/NgR signaling pathway participated in the formation of pathological features in AD. Inhibition of the Nogo/NgR signaling pathway significantly reduced the deposition of Aβ and phosphorylation of tau in APP/PS1 mice. NEP1-40, a competitive antagonist peptide of the Nogo/NgR pathway, is previously shown to promote axon regeneration and improve outcome after spinal cord injury and stroke in vivo [33, 39, 40]. Thus, NEP1-40 could effectively inhibit the Nogo/NgR pathway and is safe to use in animal models. Recent studies indicate that both Nogo and NgR contributed to the pathology of AD. For example, deleting Nogo improves cognitive function of APP transgenic mice [8] and intracerebroventricular administration of soluble NgR reduces brain Aβ plaque load [7, 12]. Additionally, Nogo receptor regulated Aβ production via interaction with APP and BACE1 and NgR2 ablation in AD mice resulted in the decrease of amyloid deposition [41]. Our results are consistent with these published data, showing that the blockage of Nogo/NgR pathway significantly attenuated amyloidogenic processing of APP and the activity of GSK3β in AD mice, which reduced Aβ deposition and phosphorylated levels of tau, respectively.

Secondly, we also verified that neuroinflammatory environment generated from the Nogo/NgR-activated BV-2 microglia induced the production of Aβ and phosphorylation of tau, which at least partially accounted for NEP1-40 function in vivo. As Nogo receptor is expressed in both neurons and microglia, we dissect what the cellular component that NEP1-40 acts by isolating microglia from neurons in vitro. Using BV-2 microglia in culture, we interfered with the expression of NgR by siRNA in the presence and absence of Nogo peptide to generate the conditioned medium with different conditions with or without Nogo/NgR activations. Using the CM from different microglia, we addressed the question of whether the CM from Nogo/NgR-activated BV-2 microglia can promote amyloidogenesis and tau hyperphosphorylation in neuronal cultures. Our data clearly demonstrated that the CM from Nogo/NgR-activated BV-2 microglia promoted Aβ production and tau hyperphosphorylation, the main pathological hallmarks of AD. These changes activated by the CM could be blocked by NgR siRNA treatment of BV-2 microglia. We confirmed that high levels of proinflammatory cytokines and gene transcripts but lower levels of anti-inflammatory cytokines and gene transcripts are present in the CM and lysates from Nogo/NgR-activated BV-2 microglia. These cytokine changes can be reversed by the treatment of microglia with siRNA of NgR. Our data indicated that the changes of the cytokine profiles in the BV-2 microglia were due to the regulation of the Nogo/NgR pathway in these microglia. Our data also indicated that primary neurons can respond to the proinflammatory factors present in CM for Aβ production and tau-phosphorylation. However, our data could not eliminate the possibility that NEP1-40 could directly act on neurons to modulate Aβ production and tau phosphorylation.

Microglia, the resident immune cells in the brain, play core roles in neuroinflammation. The activation of microglia exerts both toxic and protective effects on AD pathogenesis. Many research have proven that a lot of proinflammatory mediators enhance the expression and the amyloidogenic processing of APP to increase the production of Aβ [42]. For example, LPS could induce the release of proinflammatory cytokines including IL-1β, TNF-α, and so on and further exacerbate Aβ and tau pathology [43–45]. IL-1β and TNF-α contribute to the production of APP and its processing into Aβ in vitro and in vivo [46, 47]. Moreover, IL-4 enhances M2 phenotype in vivo and reveals a trend toward a decreased trend in Aβ deposition [48]. Furthermore, accumulating evidence links the microglia-driven neuroinflammatory responses to NFT formation and tau pathology [49]. For instance, IL-1β has been shown to increase tau phosphorylation increasing the activity of GSK-3β and MAPK [50, 51]. This evidence demonstrates that neuroinflammation is involved in AD pathogenesis. Our results showed that neuroinflammation induced by Nogo/NgR pathway activation also affected AD pathogenesis. The inhibition of the Nogo/NgR pathway improved the neuroinflammatory environment in APP/PS1 mice. The neuroinflammatory environment from Nogo/NgR pathway activation increased Aβ production and tau phosphorylation in neurons. Furthermore, Aβ aggregation termed β-amyloid fibrils is associated with AD [52, 53], and Aβ_1–42_ has been shown to aggregate into amyloid fibrils more readily than Aβ_1–40_ [54]. When treated with neuroinflammatory medium from Nogo/NgR pathway-activated BV-2 microglia, the aggregation of Aβ_1–42_ was enhanced and depolymerization of Aβ_1–42_ was decreased. After activation, microglia secrete inflammatory mediators and reactive oxygen species, which can aggravate the aggregation of Aβ in AD [55]. For example, IL-1β induces the expression of APP and increases intracellular aggregation of Aβ in human myotubes [56]. Hence, inflammatory mediators in CM from Nogo/NgR pathway-activated BV-2 microglia might aggravate the aggregation of Aβ and the mechanism need to further study. This data suggested that neuroinflammation from Nogo/NgR pathway activation in microglia participated in AD pathogenesis via modulating the balance between proinflammatory and anti-inflammatory mediators.

The expression of NgR is increased in patients with AD and aged rats with deficits of spatial cognition [9, 10]. Moreover, our previous research found that with aging, the expression of NgR on microglia was significantly increased [57], and Nogo bound with NgR on microglia increased the expression of proinflammatory cytokines [19]. In this study, we found that neuroinflammation induced by Nogo/NgR pathway in microglia could enhance the formation of Aβ plaques and hyperphosphorylation of tau. Thus, as aging, the increased expression of NgR might promote microglia become more proinflammatory, resulting in increased Aβ accumulation and occurrence and deterioration of AD. Furthermore, apart from Aβ and tau pathophysiology in AD, Nogo/NgR pathway may play roles in synaptic damage. Proinflammatory cytokines, reactive oxygen species, and neurotoxic products involve in synaptic damage in AD [58]. Thus, Nogo/NgR pathway may contribute to synaptic loss in AD directly and indirectly.

Despite several studies that imply the relationship between the Nogo, NgR, and AD, only a few detailed mechanisms have been provided to date. Two points are particularly novel contributions to this field in our study. Firstly, the Nogo/NgR signaling pathway participated in the formation of pathological features including deposition of Aβ and phosphorylation of tau in AD. Secondly, neuroinflammatory environment generated from the Nogo/NgR-activated microglia induced the production of Aβ and phosphorylation of tau, which at least partially accounted for NEP1-40 function in vivo. These results imply a better understanding of the role of Nogo/NgR signaling pathway in AD and offer a new strategy to treat AD. Small-molecular drugs and natural drugs could be investigated to target Nogo/NgR pathway, and these drugs may be new therapeutic use in AD.

Although our findings in these studies are important and reveal a critical mechanism of Nogo/NgR-activated microglia underlying inflammation-induced pathogenesis of AD, there are also limitations. It is still not known what exact factor in the CM plays a key role in the AD pathogenesis is also unknown. Whether the Nogo/NgR pathway on microglia has any effect on Aβ clearance by microglia remains unclear, and our team is working hard in this field. Future studies should focus to address these critical questions.

## Conclusions

In summary, the neuroinflammation regulated by Nogo/NgR pathway was involved in the formation of Aβ plaques and tau hyperphosphorylation in AD. The suppression of the Nogo/NgR pathway by NEP1-40 attenuated the deposition of Aβ plaque, improved tau pathology, and inhibited detrimental neuroinflammation in AD mice. Moreover, neuroinflammatory CM from Nogo/NgR-activated BV-2 microglia exacerbated the production of Aβ and phosphorylation of tau in neurons. These results suggest a better understanding of the role of neuroinflammation produced by Nogo/NgR-activated microglia on the pathogenic mechanism of AD and offer a new strategy to treat AD.

## Additional files

Additional file 1: Figure S1. The efficiency of the knockdown with NgR siRNA and the cellular ability of BV-2 microglial cells. (A and B) The expression of NgR on BV-2 microglia was determined by western blot after transfected with NgR siRNA or control siRNA. (C) After BV-2 microglia treatment with NgR siRNA or control siRNA, the survival rate of microglia was determined by MTT assay. Values were reported as mean ± SD, as a percentage of values determined in PBS group (control, 100 %). **p* < 0.05; ***p* < 0.01; ****p* < 0.001, when compared with PBS, *n* = 3. (PDF 11 kb) Additional file 2: Figure S2. The levels of secreted Aβ40 and Aβ42 in BV-2 microglia-conditioned medium. Before added to the protein-coated wells, BV-2 microglia was transfected with Ctrl siRNA or NgR siRNA to suppress the expression of NgR. The cells were then exposed to PBS or Nogo-P4 for 6 h. And then, the conditioned medium was collected and centrifuged to discard cell debris. The release of Aβ40 (Figure A) and Aβ42 (Figure B) in the conditioned medium was determined using ELISA. Values were reported as mean ± SD. *n* = 3. (PDF 95 kb)

## Abbreviations

AD: Alzheimer’s disease
ADAM10: a disintegrin and metalloproteinases 10
Arg1: arginase 1
Aβ: β-amyloid
CCL2: chemokine (C-C motif) ligand 2
CM: conditioned medium
COX-2: cyclooxygenase-2
Fizz1: found in inflammatory zone 1
IL-10: interleukin-10
IL-1β: interleukin-1β
IL-4: interleukin-4
iNOS: inducible nitric oxide synthase
NFTs: neurofibrillary tangles
NF-κB: nuclear factor-kappa B
NgR: Nogo receptor
NO: nitric oxide
PFA: paraformaldehyde
PGE2: prostaglandin E2
PHF: paired helical filament
PLL: poly-L-lysine
siRNA: small interfering RNA
STAT3: signal transducers and activators of transcription3
ThT: Thioflavin-T
ThioS: thioflavin S
TNF-α: tumor necrosis factor-α
Ym1: chitinase-like 3 (Chil3)
