# Supplementary figures and images for: The blockage of the Nogo/NgR signal pathway in microglia alleviates the formation of Aβ plaques and tau phosphorylation in APP/PS1 transgenic mice
Source: J Neuroinflammation. 2016 Mar 3;13:56. doi: 10.1186/s12974-016-0522-x (PMC4776389; doi:10.1186/s12974-016-0522-x)

# Additional file 1: Figure S1

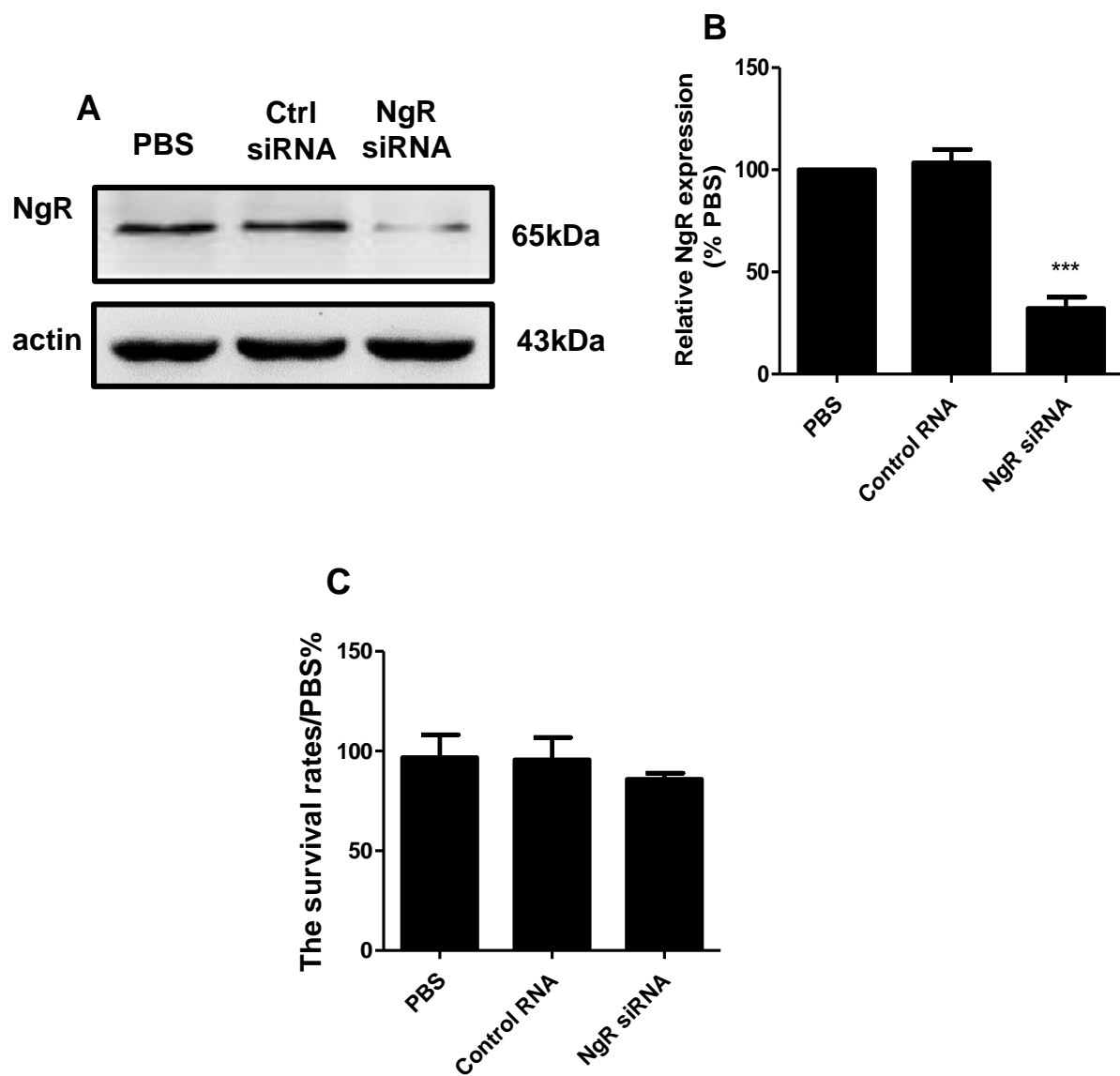

Supplement: Additional file 1: Figure S1. — The efficiency of the knockdown with NgR siRNA and the cellular ability of BV-2 microglial cells. (A and B) The expression of NgR on BV-2 microglia was determined by western blot after transfected with NgR siRNA or control siRNA. (C) After BV-2 microglia treatment with NgR siRNA or control siRNA, the survival rate of microglia was determined by MTT assay. Values were reported as mean ± SD, as a percentage of values determined in PBS group (control, 100 %). *p < 0.05; **p < 0.01; ***p < 0.001, when compared with PBS, n = 3. (PDF 11 kb) [file 12974_2016_522_MOESM1_ESM.pdf]

## Additional file 2: Figure S2

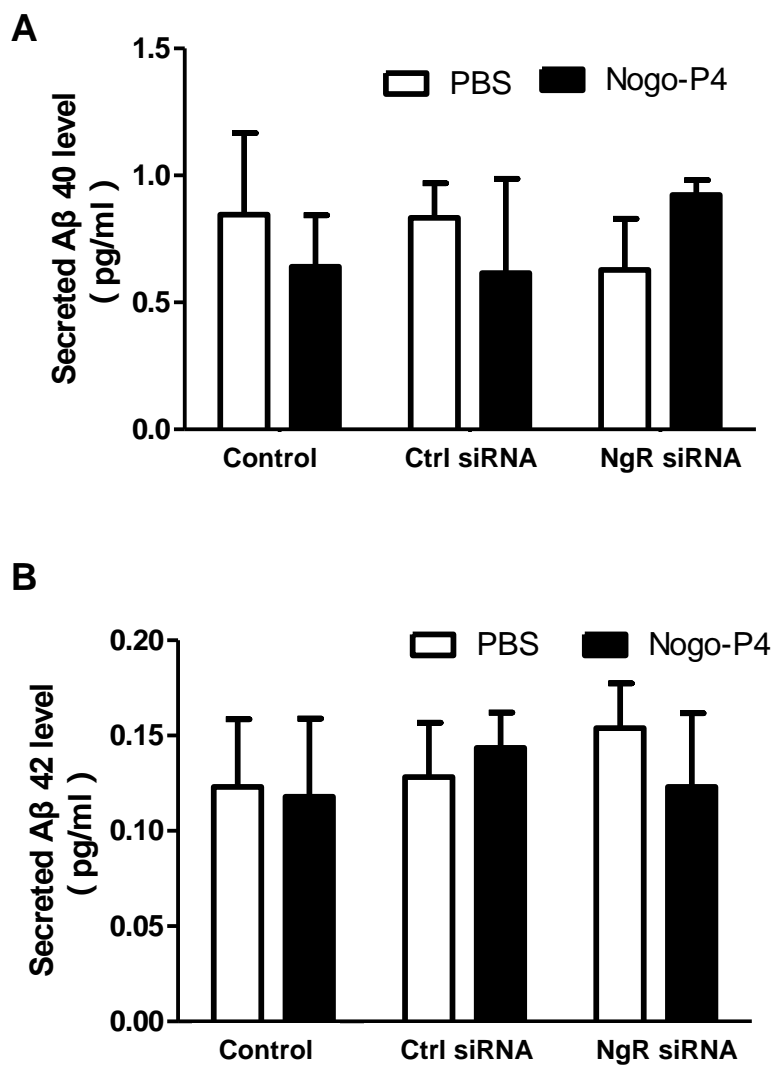

Supplement: Additional file 2: Figure S2. — The levels of secreted Aβ40 and Aβ42 in BV-2 microglia-conditioned medium. Before added to the protein-coated wells, BV-2 microglia was transfected with Ctrl siRNA or NgR siRNA to suppress the expression of NgR. The cells were then exposed to PBS or Nogo-P4 for 6 h. And then, the conditioned medium was collected and centrifuged to discard cell debris. The release of Aβ40 (Figure A) and Aβ42 (Figure B) in the conditioned medium was determined using ELISA. Values were reported as mean ± SD. n = 3. (PDF 95 kb) [file 12974_2016_522_MOESM2_ESM.pdf]
